# Diagnosing virtual patients: the interplay between knowledge and diagnostic activities

**DOI:** 10.1007/s10459-023-10211-4

**Published:** 2023-04-13

**Authors:** Maximilian C. Fink, Nicole Heitzmann, Victoria Reitmeier, Matthias Siebeck, Frank Fischer, Martin R. Fischer

**Affiliations:** 1 Institute of Medical Education, University Hospital, LMU Munich, Munich, Germany; 2 Department for Education, University of the Bundeswehr Munich, Institute of Education, Learning and Teaching with Media, Werner-Heisenberg-Weg 39, 85577 Neubiberg, Germany; 3 Department of Psychology, LMU Munich, Munich, Germany; 4 Munich Center of the Learning Sciences (MCLS), LMU Munich, Munich, Germany

**Keywords:** Clinical reasoning, Diagnostic activities, Diagnostic process, Knowledge, Virtual patients, Simulation

## Abstract

Clinical reasoning theories agree that knowledge and the diagnostic process are associated with diagnostic success. However, the exact contributions of these components of clinical reasoning to diagnostic success remain unclear. This is particularly the case when operationalizing the diagnostic process with diagnostic activities (i.e., teachable practices that generate knowledge). Therefore, we conducted a study investigating to what extent knowledge and diagnostic activities uniquely explain variance in diagnostic success with virtual patients among medical students. The sample consisted of *N* = 106 medical students in their third to fifth year of university studies in Germany (6-years curriculum). Participants completed professional knowledge tests before diagnosing virtual patients. Diagnostic success with the virtual patients was assessed with diagnostic accuracy as well as a comprehensive diagnostic score to answer the call for more extensive measurement of clinical reasoning outcomes. The three diagnostic activities hypothesis generation, evidence generation, and evidence evaluation were tracked. Professional knowledge predicted performance in terms of the comprehensive diagnostic score and displayed a small association with diagnostic accuracy. Diagnostic activities predicted comprehensive diagnostic score and diagnostic accuracy. Hierarchical regressions showed that the diagnostic activities made a unique contribution to diagnostic success, even when knowledge was taken into account. Our results support the argument that the diagnostic process is more than an embodiment of knowledge and explains variance in diagnostic success over and above knowledge. We discuss possible mechanisms explaining this finding.

**Supplementary Information:**

The online version contains supplementary material available at 10.1007/s10459-023-10211-4.

## Introduction

A vast majority of researchers agree that clinical reasoning is an extensive construct that consists of a variety of key components, including knowledge, diagnostic processes, and outcome measures (Elstein, 2009; Schmidt et al., 1990; Young et al., 2018). Existing research has investigated each of these key components of clinical reasoning, but the exact contribution of the diagnostic process to outcome measures, such as diagnostic accuracy, and its interaction with knowledge remains largely unexplored. This paper adds to research on the associations of the key components of clinical reasoning by examining the interplay between knowledge and diagnostic activities. Diagnostic activities were used as an operationalization of the diagnostic process because they can be considered teachable practices that generate knowledge (Heitzmann et al., 2019). Research on the relationships between the aforementioned key components of clinical reasoning can contribute to improving our theoretical understanding of this construct. Moreover, research carried out on this topic can yield relevant insights for assessment methods and using instructional support in medical education (Daniel et al., 2019; Heitzmann et al., 2017).

### Three main perspectives on clinical reasoning

Three main perspectives on clinical reasoning can be distinguished. First, knowledge-centered theories, such as illness script theory (Schmidt et al., 1990), assume that the amount, type, and structure of knowledge networks developed through formal training and practical medical experience are crucial for diagnosing in an automatic pattern recognition process (Charlin et al., 2007). Second, problem-solving theories, typically emphasizing the hypothetico-deductive method (Elstein et al., 1978, 1990), argue that reasoning strategies (also called diagnostic processes), such as generating hypotheses, play an important role in diagnosing in a conscious way. Third, cognitive theories suppose that diagnosing is heavily influenced by biases and the interplay between different cognitive systems (Elstein & Schwartz, 2002). A popular example of cognitive theories are medical dual-process theories of diagnosing (Croskerry, 2009; Eva, 2004; Evans, 2008). These theories assume that a separate fast, unconscious system and slow, conscious cognitive system are both involved in diagnosing. However, the three described theoretical perspectives are no longer considered mutually exclusive and most researchers concur that clinical reasoning includes aspects of knowledge, biases, and diagnostic processes to some extent (Eva, 2004).

### Assessing clinical reasoning with virtual patients

Virtual patients can be defined as digital simulations of important clinical situations such as the medical interview providing some kind of interactivity and containing audiovisual materials (Cook et al., 2010). Moreover, virtual patients are conducted in a highly standardized way and can offer detailed log data about participants’ diagnostic processes. Perhaps for these reasons, virtual patients have become increasingly popular tools for formative and summative assessment in medical education in recent decades (Boulet & Durning, 2019; Ryall et al., 2016). The aforementioned features and their widespread use highlight that virtual patients could be particularly suitable for investigating the relationships among the key components of clinical reasoning.

### Focused and comprehensive outcome measures used in virtual patients

In the past, most virtual patient assessments used the focused outcome measure of diagnostic accuracy (Daniel et al., 2019), which can be defined as the correctness of the final diagnosis. Diagnostic accuracy has the advantage of being rather easy to measure electronically and can be scored relatively objectively. However, practitioners and researchers have repeatedly argued that virtual patients should capture diagnostic success more comprehensively (Daniel et al., 2019; Elder, 2018; Round et al., 2009). Comprehensive outcome measures for virtual patients can include but are not limited to additional diagnostic tests, treatment decisions, prognosis, and justifications for all of these aspects (Daniel et al., 2019). Incorporating aspects like these into virtual patient assessments could help to diminish overtreatment and undertreatment of patients (Mamede & Schmidt, 2014) and gain more detailed insights into students’ specific errors in diagnosing.

### Heitzmann’s framework of clinical reasoning

Our study operationalizes clinical reasoning based on a framework by Heitzmann et al. (2019) and related literature (Förtsch et al., 2018; Stark et al., 2011). In terms of the three aforementioned perspectives on clinical reasoning, this framework provides a problem-solving theory that also incorporates knowledge-related aspects. Knowledge is assessed as *professional knowledge,* consisting of conceptual and strategic knowledge. *Conceptual knowledge* is knowledge about facts and constructs, termed “knowing what”, whereas *strategic knowledge* refers to knowledge about possible paths and heuristics in diagnosing, termed “knowing how” (Förtsch et al., 2018; Stark et al., 2011). The diagnostic process is operationalized in this framework via eight diagnostic activities. *Diagnostic activities* are knowledge-generating practices that are learned through training. They can occur in varying quantity, quality, and sequence—but it is mainly their quality that is assumed to be linked with diagnostic success (Heitzmann et al., 2019). The three diagnostic activities of hypothesis generation, evidence generation, and evidence evaluation (Heitzmann et al., 2019) were selected because theoretical accounts and empirical studies indicate that they are related to diagnostic success in the context of medical history-taking (Fink et al., 2022; Ramsey et al., 1998; Roter & Hall, 1987). *Hypothesis generation* is defined as creating a case diagnosis based on initial key information about the patient. *Evidence generation* refers to gathering and creating additional information for the diagnosis. *Evidence evaluation* is described as interpreting the meaning and reliability of pieces of acquired information. Diagnostic success can be measured in line with this framework with a focused *diagnostic accuracy score* and a *comprehensive diagnostic score*.

### The relationships among the key components of clinical reasoning

#### The relationship between prior professional knowledge and diagnostic success

Stark et al. (2011) investigated the associations of conceptual knowledge, strategic knowledge, and performance on text-based problem-solving tasks, focusing on diagnostic accuracy, in a sample of medical students. Diagnostic success in the problem-solving tasks was positively correlated with conceptual and strategic prior knowledge. Adding to these results, a study by Schmidmaier et al. (2013) with medical students as participants examined associations between prior knowledge and performance in a text-based problem-solving task that required clinical decision-making. The study found a high correlation with strategic knowledge and a medium correlation with conceptual knowledge for the problem-solving task. Recently, associations between knowledge and diagnostic success have also been found in the context of virtual patients. In a study by Kiesewetter et al. (2020), medical students completed knowledge tests and virtual patient assessments. Participants with a high combined score for conceptual and strategic knowledge performed better in diagnostic accuracy than participants with low scores in the knowledge test.

#### The relationship between diagnostic activities and diagnostic success

Associations between the quality of hypothesis generation and diagnostic success have been found in a study in which participants solved text-based cases (Coderre et al., 2010). Moreover, correlations between hypothesis generation and diagnostic success measures have been discovered with standardized patients (Barrows et al., 1982; Neufeld et al., 1981). Taken together, these studies suggest that the quality of hypothesis generation is positively associated with diagnostic success in other contexts as well, such as with virtual patients.

Correlations between the quality of evidence generation and diagnostic success have also been reported. Woolliscroft et al. (1989) investigated physicians’ history-taking with standardized patients and found an association between specific questions asked and the percentage of critical features obtained. In a study by Stillman et al. (1991), physicians took part in standardized patient evaluations. Performance on a history-taking checklist filled out by the standardized patients had a small but significant positive correlation with achieved diagnostic accuracy. Moreover, Fink et al. (2021b) discovered a medium positive association between the quality of evidence generation and diagnostic accuracy in virtual patients.

A relationship between the quality of evidence evaluation and diagnostic success can also be presumed. The data interpretation process that takes place within the script concordance test (Charlin et al., 2000), a valid and reliable test of clinical reasoning, shares similarities with the definition of evidence evaluation by Heitzmann et al. (2019). Investigating such a data interpretation process in virtual patients rather than the text-based cases included in the script concordance test seems particularly promising.

Up to now, the contribution of diagnostic activities to diagnostic success has not been sufficiently researched by studies investigating multiple predictors together—with one notable exception. Groves et al. (2003) examined failures in three diagnostic processes when working on text-based cases in medicine. Two of these diagnostic processes were similar to the diagnostic activities of hypothesis generation and evidence evaluation. The study found that failures in these diagnostic processes predicted lack of diagnostic success (Groves et al., 2003).

#### Are diagnostic activities an embodiment of knowledge?

As previously mentioned, an analysis of whether diagnostic activities make a unique contribution to explaining diagnostic success over and above knowledge seems warranted. This is also the case because the reported studies on the interplay of diagnostic activities and diagnostic success did not systematically control for prior knowledge. Two possible mechanisms explain the relationship between knowledge, the diagnostic process, and diagnostic success: (1) Prior knowledge is the sole predictor of diagnostic success. This mechanism is supported by illness-script theory (Schmidt et al., 1990), which would consider diagnostic activities an embodiment or manifestation of knowledge. (2) Prior knowledge and diagnostic activities have both unique contributions to diagnostic success. This mechanism relies on the notion that diagnostic processes build on, but are not entirely determined by accessible knowledge (Norman, 2005). At this point, it should have become clear that this study focusses on medical students who still primarily diagnose consciously. Our considerations do not extend to medical experts who possess deep and rich knowledge networks and employ a quick and automatic pattern recognition process (Schmidt & Rikers, 2007).

## Research question and hypotheses

This study investigates to what extent diagnostic activities and prior professional knowledge uniquely explain variance in diagnostic success. This research question is examined for two indicators of diagnostic success: a comprehensive diagnostic score and diagnostic accuracy. Concerning comprehensive diagnostic score, we hypothesize that three diagnostic activities (H1.1), namely hypothesis generation, evidence generation, and evidence evaluation, as well as prior professional knowledge (H1.2), consisting of conceptual and strategic knowledge, both explain variance. Moreover, we assume that the diagnostic activities increase the amount of explained variance over and above prior professional knowledge (H1.3). For diagnostic accuracy, we propose the same hypotheses as for comprehensive diagnostic score (H2.1–H2.3).

## Method

### Procedure, recruitment and participants

The participants began the study by completing a conceptual and a strategic knowledge test. Then, the participants underwent a familiarization procedure explaining how to work with the virtual patients. Afterward, the participants diagnosed multiple virtual patients on the topic of history-taking for dyspnea.

We recruited students from the Medical Faculty of LMU Munich as participants of our study from October 2019 to February 2021 by advertising online, via e-mail and in courses. Participation was open for students in their third to fifth year of medical school (with a 6-year program) fluent in German. Moreover, participation was voluntary and reimbursed with €10 per hour. As e-mails were sent to all students fulfilling the eligibility criteria, we believe that an audience of about *N* = 1650 medical students between year three and five of LMU medical school was approached.

Altogether, *N* = 121 medical students took part in the study. Due to using hierarchical regression analyses and for consistency reasons, participants with missing values on key variables were dropped, resulting in a final sample of *N* = 106 participants, with a mean age of *M* = 24.76 years, SD = 3.83. This final sample included *n* = 70 females (66.0%), *n* = 9 males (8.5%) and *n* = 27 (25.5%) participants without gender information. This high percentage of participants without gender information was probably primarily caused by an electronic form that allowed participants to skip this question. The described results suggest the possibility that our sample was not fully representative with respect to gender. More specifically, the proportion of males in our sample might have been lower than at the Medical Faculty of LMU where they make up about 30% of all medical students.

Concerning participants’ prior experience, it should be mentioned that they were studying medicine based on a hybrid curriculum with strong problem-based components. The participants were used to problem-based learning with tutorial cases and case-based learning in various formats including virtual patients. Moreover, they were familiar with engaging in basic clinical tasks like history-taking, carrying out physical examinations, and case presentations at the bedside and in the classroom. The curriculum at LMU is relatively flexible. Some medical students selected a module called *respiratory diseases* related to dyspnea in year three while other medical students took the same module in year four or even five. About 2/3 of students had taken this module before taking part in our study whereas about 1/3 had not taken this module prior to participation. To take this point into consideration, we examined the contribution of knowledge and the diagnostic process separately for medical students who took part or did not take part in the module on respiratory diseases in the Appendix.

### Knowledge tests

#### Conceptual knowledge test

The conceptual knowledge test consisted of previously validated exam questions taken from an electronic item bank used by several medical faculties (UCAN Assessment Network., 2019). To find suitable items, we used the following approach: (1) We screened the item bank for questions related to dyspnea and history-taking for dyspnea patients, (2) We considered only questions with good face validity which were evaluated on medical students between years three and five with a decent sample, (3) We selected 20 questions with varying levels of difficulty that did not possess extreme difficulty scores (0.10 ≥ M ≤ 0.90). Moreover, we ensured that the majority of selected questions had a medium level of difficulty, but also easy and difficult questions were included. A prior version of the used knowledge test was validated in a study on medical students with comparable expertise and displayed a medium positive correlation with diagnostic accuracy (Reitmeier, 2020). The questions used two popular question formats: single-choice questions and multiple-response questions. In single-choice questions, 1.0 points were allocated for the correct answer. In multiple-response questions, points were awarded as follows: 1.0 points were given for an entirely correct answer pattern, and 0.50 points were allocated if more than 50% of the participant’s answers were correct (Bauer et al., 2011). To build a scale, the number of points achieved was divided by the number of questions posed. This scale ranged from 0 (*low knowledge*) to 1 (*high knowledge*). The test reached acceptable reliability of $\alpha$ = 0.66.

#### Strategic knowledge test

Strategic knowledge was measured with four key feature cases (Hrynchak et al., 2014) that centered around knowledge of dyspnea and history taking for dyspnea patients. The strategic knowledge test was created by a general practitioner and validated in a prior study, which reported a medium positive correlation of this instrument with diagnostic accuracy in virtual patients (Reitmeier, 2020). Each key feature case contained four single-choice questions. These four single-choice questions focused on the diagnosis, treatment, symptoms, and further diagnostic measures. 1.0 points were allocated for each correct answer. The scale for the strategic knowledge test was built by dividing the number of points achieved by the number of questions posed. This scale ranged from 0 (*low knowledge*) to 1 (*high knowledge*). The scale’s reliability was acceptable, with $\alpha$ = 0.65.

### Virtual patients

#### Topic and simulation scenario

**Fig. 1:**
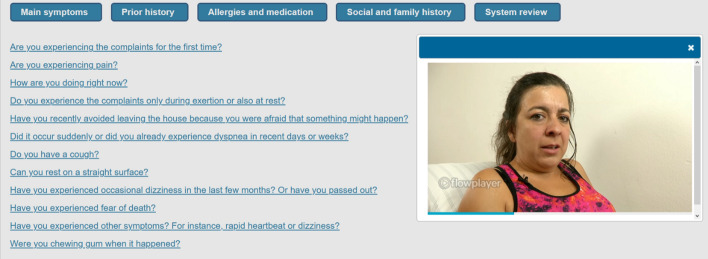
Screenshot of a virtual patient

The participants encountered multiple virtual patients representing different causes of dyspnea and engaged in history-taking for diagnosing. The simulation scenario for the virtual patients was as follows. The simulation began with the presentation of prior information (e.g., lab results) and the patient’s chief complaint. Next, participants selected questions to ask the virtual patient from a menu of history-taking questions. This menu included up to 69 standardized questions for each case and was subdivided into the categories *main symptoms*, *prior history*, *allergies and medication*, *social and family history*, and *system review*. The history-taking questions and menu had been validated in previous studies (Fink et al., 2021a, 2021b), and examples of the history-taking questions are listed in Appendix S1. After the participant selected a question from the menu, the corresponding answer was streamed as a video. Each virtual patient encounter lasted between a minimum of 5 minutes and a maximum of ten minutes. Before each virtual patient, participants were instructed to spend at least the minimum amount of time working with the simulation. They were then notified by prompts when the minimum and maximum time had been reached. A screenshot of a virtual patient at the point of selecting questions from the menu is provided in Fig. 1.

#### Creation of the virtual patients and electronic assessment environment

As a first step to creating the virtual patients, professional actors were hired and then trained for their role by a physician and an acting coach. When filming the videos, the professional actors exhibited the patients’ symptoms according to their script. After editing, the videos were integrated with additional case information to create the virtual patients in the electronic assessment environment CASUS (Instruct, 2021).

#### Diagnostic success measures

Diagnostic success was assessed with *diagnostic accuracy* and a *comprehensive diagnostic score*.

**Fig. 2:**
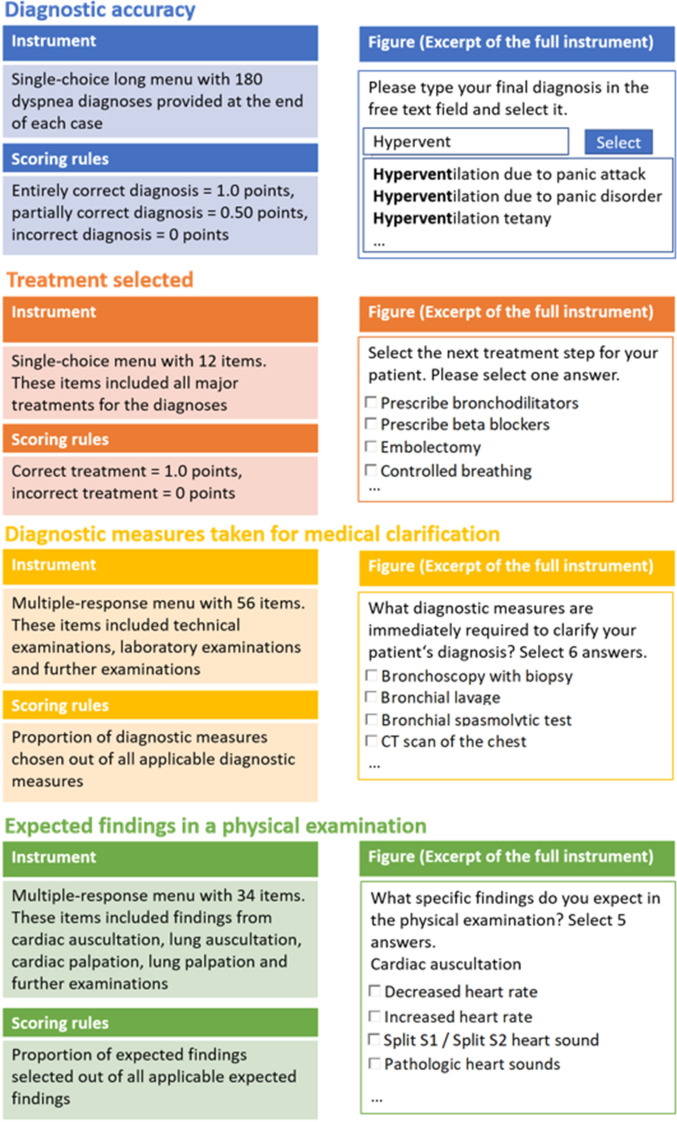
Diagnostic success measures

Diagnostic accuracy was assessed with a long menu that consisted of 180 dyspnea-related diagnoses. A long menu is a free text field with a concealed list of answers and an autocomplete feature in which one answer can be selected. The solutions used to score the answers were determined by a licensed physician and a specialist in general medicine and previously used in a study by Fink et al. (2021b). More information on the instrument is available in Fig. 2.

The comprehensive diagnostic score encompassed four equally-weighted variables: (1) *diagnostic accuracy*, (2) *treatment selected*, (3) *diagnostic measures taken for medical clarification,* and (4) *expected findings in a physical examination*. Diagnostic accuracy was operationalized and measured as previously described. Treatment selected was defined as the most important, next treatment for the patient. Diagnostic measures taken for clarification refer to all technical/diagnostic measures immediately necessary to investigate the diagnosis further. Expected findings in the physical examination denote the specific signs and symptoms expected to be observed in a physical exam following history-taking. More details on the instruments and the scoring are provided in Fig. 2. Participants’ responses to the described variables were compared to a sample solution jointly developed by a licensed physician and a specialist in general medicine using *R* scripts. A principal component analysis with varimax rotation as well as corresponding Eigenvalue and scree plot analyses indicated that all four variables belong to one comprehensive diagnostic score factor and explained 61.1% of the variance in comprehensive diagnostic score (see Appendix S2). Due to the different answer formats and points allocated, scores on the four variables were standardized before calculating the average comprehensive diagnostic score.

#### Case selection and preliminary analyses

The diagnoses for the four virtual patient cases included in our study and the respective descriptive statistics for these cases are reported in Table 1. It should be mentioned that two other cases had to be excluded from our study due to floor effects on diagnostic success measures. Please see Appendix S3 for the diagnoses and descriptive statistics for these excluded cases.

**Table 1:** Descriptive statistics for diagnostic activities and diagnostic success measures

	Case 1	Case 2	Case 3	Case 4	Total
	*M (SD)*	*M (SD)*	*M (SD)*	*M (SD)*	*M (SD)*
*Diagnostic activities*					
Hypothesis generation	0.35 (0.26)	0.73 (0.41)	0.08 (0.20)	0.23 (0.39)	0.35 (0.19)
Evidence generation	0.40 (0.16)	0.37 (0.20)	0.67 (0.24)	0.32 (0.20)	0.44 (0.13)
Evidence evaluation	0.59 (0.21)	0.48 (0.19)	0.37 (0.25)	0.50 (0.23)	0.49 (0.11)
*Diagnostic success measures*					
Diagnostic accuracy	0.31 (0.33)	0.64 (0.46)	0.49 (0.47)	0.32 (0.41)	0.43 (0.23)
Treatment selected	0.60 (0.49)	0.69 (0.47)	0.50 (0.50)	0.44 (0.50)	0.56 (0.25)
DM	0.56 (0.17)	0.59 (0.37)	0.42 (0.22)	0.36 (0.30)	0.48 (0.17)
EF	0.52 (0.16)	0.56 (0.21)	0.47 (0.15)	0.78 (0.35)	0.58 (0.14)
*Comprehensive*					
diagnostic score	–	–	–	–	0.04 (1.00)

*Case 1* Hypertrophic cardiomyopathy, *Case 2* Pneumonia, *Case 3* Pulmonary embolism with a coagulation disorder, *Case 4* Panic attack

The comprehensive diagnostic score was normalized with z-scores from − 3 to + 3 and only calculated for the total score. Range of all other variables: (0) *low* to (1) *high*. Abbreviations: DM = Diagnostic measures taken for medical clarification, EF = Expected findings in a physical examination

#### Diagnostic activities

**Fig. 3:**
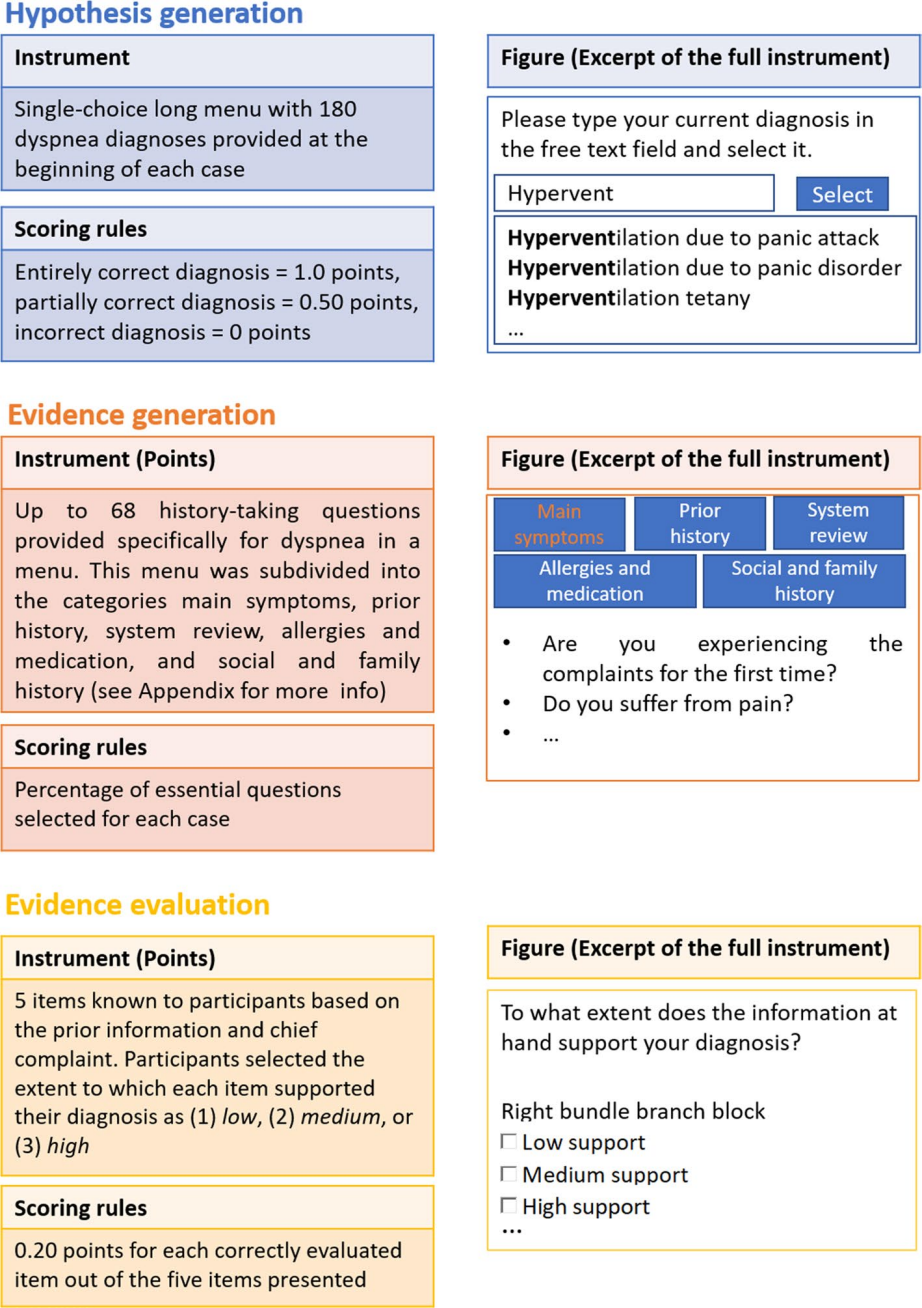
Diagnostic activities measures

Based on Heitzmann et al. (2019), we also assessed three diagnostic activities. We measured the quality of *hypothesis generation* using the same long menu previously described as an instrument for measuring diagnostic accuracy. This means that one of 180 diagnoses was selected here as well by the learner. In contrast to diagnostic accuracy, the measure of hypothesis generation occurred at the beginning of each virtual patient encounter. The quality of *evidence generation* was assessed based on the questions selected during history-taking. Participants selected these questions from the menu described in Appendix S1, and all questions were specific to dyspnea and standardized across the virtual patients. To score this variable, we used a coding scheme previously utilized for the same history-taking questions on the same virtual patients by Fink et al. (2021b). This coding scheme was a joint, common solution developed by one licensed physician and one specialist in general medicine that specified the essential questions for each case. The quality of *evidence evaluation* was measured retrospectively after the participant diagnosed each virtual patient. In completing this instrument, participants judged to what extent aspects known from the prior information and chief complaint supported their final diagnosis for the virtual patient. This instrument and the corresponding sample solutions were newly developed by a licensed physician. Content validity and correctness of the instrument and solutions were reviewed by another physician who was a specialist in general medicine. As this instrument was used for the first time, we cannot report external validity measures on it. Additional information on all three diagnostic activities is provided in Fig. 3. It should be added that the participants’ diagnostic activities were automatically compared to the sample solutions via *R* scripts.

### Data collection method and statistical and power analyses

The study’s data was gathered from October 2019 until February 2021 at the University Hospital, LMU Munich, in Germany. Due to the COVID-19 pandemic, the data collection method had to be changed while the study was running. Until March 2020, data from* n* = 30 participants included in the final sample was gathered on-site in a computer lab. After March 2020, data from *n* = 76 participants in the final sample was collected web-based. A control analysis reported in Appendix S4 showed that the lab-based and web-based participants differed in terms of knowledge, diagnostic activities, and diagnostic success variables. Therefore, we ran statistical tests for effects of the data collection method in Appendix S5 by repeating the regression analyses reported in the results section while including the data collection method as a factor. In these analyses, we modeled interaction effects between the data collection method and all relevant predictors and found that the effect of the predictors did not depend on the data collection method.

We used R version 4.0.2 (R Core Team, 2020) for our statistical analyses. Multiple regression and hierarchical regression analyses were conducted to investigate our research questions. Frequently used assumptions checks for regression models, including residuals vs. fitted values plots, Q–Q plots, and scale-location plots, confirmed that these regression models were a good fit. In all statistical analyses, the significance level was set to $\alpha$ = 0.05.

Post hoc power analyses were conducted with G*Power version 3.1 (Faul et al., 2009). For the power analyses, we set the error probability to $\alpha$ = 0.05 and the sample size to *N* = 106. Our analyses were based on a medium effect of Cohen’s f^2^ = 0.15 and revealed power of at least $\beta$ = 0.87 for each analysis.

## Results

### Descriptive statistics and intercorrelations

Participants reached a medium score on the conceptual knowledge (*M* = 0.54, *SD* = 0.14) and strategic knowledge (*M* = 0.50, *SD* = 0.14) tests preceding the virtual patient cases. As reported in Table 1, performance in case 1 to case 4 on the diagnostic activities and diagnostic success measures was medium and can be considered suitable.

Intercorrelations for professional knowledge, the three diagnostic activities, and diagnostic success measures are reported in Table 2. The relationships between these variables are examined in more detail in the following regression models. It should be added that we found a medium correlation between conceptual and strategic knowledge (*r* = 0.55). This correlation was examined more closely for multicollinearity issues using the variance inflation index. As collinearity between the two knowledge types was slight to moderate (VIF = 1.44), both variables were included together in the regression models.

**Table 2:** Intercorrelations of knowledge, diagnostic activities, and diagnostic success measures

	1	2	3	4	5	6	7
1. Conceptual knowledge	–						
2. Strategic knowledge	0.55***	–					
3. Hypothesis generation	0.00	− 0.07	–				
4. Evidence generation	0.31**	0.47***	− 0.02	–			
5. Evidence evaluation	0.01	0.17	0.18	0.11	–		
6. Comprehensive diagnostic score	0.36***	0.41***	0.30**	0.42***	0.35***	–	
7. Diagnostic accuracy	0.23*	0.21*	0.41***	0.22*	0.18	0.76***	–

Two-tailed Pearson correlations. Note that the scores for hypothesis generation, evidence generation, evidence evaluation, the comprehensive diagnostic score, and diagnostic accuracy were aggregated over four virtual patients

*p < 0.05, **p < 0.01, ***p < 0.001

### The contribution of diagnostic activities and professional knowledge to the comprehensive diagnostic score

Regression analyses for the comprehensive diagnostic score as criterion were conducted (see Table 3). Model 1, containing diagnostic activities as predictors, was significant. As expected in H1.1, the three diagnostic activities together explained a substantial amount of variance in the comprehensive diagnostic score. Model 2a, encompassing the two aspects of professional knowledge as predictors, was also significant. In line with H1.2, professional knowledge accounted for substantial amounts of variance in the comprehensive diagnostic score. Model 2b consisted of the predictors in Model 2a plus the three diagnostic activities added in a second step; this model was also significant. A comparison of the two models indicated that Model 2b explained substantially more variance than Model 2a (*F*(3, 100) = 12.30, *p* < 0.001, $\Delta$ *R*^2^ = 0.21, $\Delta \mathrm{Adj}.$ *R*^2^ = 0.20). This finding supports H1.3, that the diagnostic activities increase the amount of explained variance in the comprehensive diagnostic score over and above professional knowledge. Additional analyses examining this research question depending on whether subjects participated in a respiratory module are provided in Appendix S6.

**Table 3:** Regression analyses for comprehensive diagnostic score as outcome

Predictor	*b*	*ß*	*ß* 95% CI	*p*	*Model test and fit*
**Model 1**					*F*(3, 102) = 17.21, *p* < 0.001
Intercept	− 2.30***			< 0.001	*R*^*2*^ = 0.34
Hypothesis generation	1.09**	0.26	[0.10, 0.42]	0.002	Adj. *R*^2^ = 0.32
Evidence generation	2.40***	0.40	[0.24, 0.56]	< 0.001	
Evidence evaluation	1.76**	0.26	[0.10, 0.42]	0.002	
**Model 2a**					*F*(2, 103) = 12.55, *p* < 0.001
Intercept	− 1.42***			< 0.001	*R*^*2*^ = 0.20
Conceptual knowledge	1.07	0.19	[− 0.02, 0.40]	0.074	Adj. *R*^2^ = 0.18
Strategic knowledge	1.66**	0.31	[0.10, 0.52]	0.005	
**Model 2b**					*F*(5, 100) = 14.05, *p* < 0. 001
Intercept	− 2.92***			< 0.001	*R*^*2*^ = 0.41
Conceptual knowledge	1.07*	0.19	[0.01, 0.37]	0.043	Adj. *R*^2^ = 0.38
Strategic knowledge	0.86	0.16	[− 0.04, 0.36]	0.121	
Hypothesis generation	1.14***	0.27	[0.12, 0.43]	< 0.001	
Evidence generation	1.60**	0.27	[0.09, 0.44]	0.003	
Evidence evaluation	1.65**	0.24	[0.08, 0.40]	0.003	

Model 1 is a multiple regression containing diagnostic activities variables. Model 2 is a hierarchical regression consisting of knowledge variables in Model 2a and knowledge and diagnostic activities in Model 2b. *b* represents unstandardized regression weights. *ß* represents standardized regression weights. CI = confidence interval. *p < 0.05, **p < 0.01, ***p < 0.001

### The contribution of diagnostic activities and professional knowledge to diagnostic accuracy

Regression analyses for diagnostic accuracy as the criterion were also conducted (see Table 4). Model 3, containing diagnostic activities as predictors, was significant. As expected in H2.1, the three diagnostic activities together explained a substantial amount of variance in diagnostic accuracy. Model 4a, encompassing conceptual and strategic knowledge as predictors, was also significant, but this was only due to a significant intercept term. However, the bivariate relations between conceptual and strategic knowledge and diagnostic accuracy were significant (see Table 2). These findings can be seen as mixed evidence for H2.2 that professional knowledge is associated with diagnostic accuracy. Model 4b consisted of the predictors in Model 4a plus the three diagnostic activities added in a second step; this model was also significant. A comparison of the two models indicated that Model 4b explained substantially more variance than Model 4a (*F*(3, 100) = 8.85, *p* < 0.001, $\Delta$*R*^2^ = 0.20, $\Delta \mathrm{Adj}.$ *R*^2^ = 0.18). This finding supports H2.3, that the diagnostic activities increase the amount of explained variance in diagnostic accuracy over and above professional knowledge. Complementary analyses investigating this research question considering participants’ completion of a respiratory module are provided in Appendix S6.

**Table 4:** Regression analyses for diagnostic accuracy as outcome

Predictor	*b*	*ß*	*ß* 95% CI	*p*	*Model test and fit*
**Model 3**					*F*(3, 102) = 10.00, *p* < 0.001
Intercept	0.00			.981	*R*^*2*^ = 0.23
Hypothesis generation	0.48**	0.40	[0.22, 0.57]	< .001	Adj. *R*^2^ = 0.20
Evidence generation	0.40*	0.22	[0.05, 0.40]	0.012	
Evidence evaluation	0.17	0.09	[− 0.09, 0.26]	0.331	
**Model 4a**					*F*(2, 103) = 3.42, *p* = 0.037
Intercept	0.19*			0.040	*R*^*2*^ = 0.06
Conceptual knowledge	0.27	0.16	[− 0.06, 0.39]	0.157	Adj. *R*^2^ = .04
Strategic knowledge	0.19	0.12	[− 0.11, 0.35]	0.302	
**Model 4b**					*F*(5, 100) = 6.99, *p* < 0.001
Intercept	− 0.12			0.335	*R*^*2*^ = 0.26
Conceptual knowledge	0.24	0.14	[− .006, 0.35]	0.172	Adj. *R*^2^ = 0.22
Strategic knowledge	0.12	0.07	[− 0.15, 0.30]	0.516	
Hypothesis generation	0.49	0.40	[0.23, 0.58]	< 0.001	
Evidence generation	0.26	0.15	[− 0.05, 0.34]	0.142	
Evidence evaluation	0.16	0.08	[− 0.10, 0.26]	0.368	

Model 3 is a multiple regression containing diagnostic activities variables. Model 4 is a hierarchical regression, consisting of knowledge variables in Model 4a and knowledge and diagnostic activities in Model 4b. *b* represents unstandardized regression weights. *ß* represents standardized regression weights. CI = confidence interval. *p < 0.05, **p < 0.01, ***p < 0.001

## Discussion

### Principal findings

#### The contribution of the diagnostic activities to diagnostic success

The diagnostic activities of hypothesis generation, evidence generation, and evidence evaluation together accounted for a substantial amount of the variance in the comprehensive diagnostic score and the focused diagnostic accuracy score (Model 1 *R*^2^ = 0.34 resp. Model 3 *R*^2^ = 0.23).

Next, we will discuss the contribution of the individual diagnostic activities. Hypothesis generation was a strong predictor of the comprehensive diagnostic score and diagnostic accuracy in both regression models (Model 1 and Model 3). This finding concurs with research highlighting the associations between hypotheses and diagnostic success in solving text-based cases (Coderre et al., 2010), as well as in standardized patients (Barrows et al., 1982; Neufeld et al., 1981). Likewise, evidence generation predicted the comprehensive diagnostic score and focused diagnostic accuracy score. This result is in line with correlational results gathered in virtual patients, standardized patients, and real-life professional contexts (Fink et al., 2021b; Stillman et al., 1991; Woolliscroft et al., 1989). Evidence evaluation, however, was only a significant predictor of the comprehensive diagnostic score, not of the diagnostic accuracy score. This unexpected result may be explained by looking at the information upon which the evidence evaluation instrument was based. In our evidence evaluation instrument, participants retrospectively assessed the extent to which five key pieces of information supported their final hypothesis. Competence in interpreting the meaning of key pieces of information and the information itself may have helped participants request the treatments and diagnostic measures included in the comprehensive diagnostic score. However, competence in interpreting the meaning of key information and the information itself may not have substantially assisted participants in selecting the correct final diagnosis.

Overall, our results demonstrate that diagnostic activities account for variance in diagnostic success measures. This result is consistent with theories that view clinical reasoning as a problem-solving process (Elstein et al., 1978, 1990), and adds to the study by Groves et al. (2003), which found that failures in diagnostic processes relatively similar to diagnostic activities predicted lack of diagnostic success. Moreover, our results suggest that diagnostic activities could serve as a fruitful starting point for providing instructional support. Instructional support in the form of prompts and other cognitively-stimulating interventions (Chernikova et al., 2019, 2020) that target diagnostic activities could potentially be effective due to the observed association between diagnostic activities and diagnostic success.

#### The contribution of professional knowledge to diagnostic success

Professional knowledge explained a substantial amount of variance in the comprehensive diagnostic score and little variance in the diagnostic accuracy score (Model 2a *R*^2^ = 0.20 resp. Model 4a *R*^2^ = 0.06).

The result that professional knowledge is predictive of comprehensive diagnostic score is in line with several empirical studies that found associations between knowledge and diagnosing in text-based problem-solving tasks and diagnosing virtual patients (Kiesewetter et al., 2020; Schmidmaier et al., 2013; Stark et al., 2011). To be more specific, we found in Model 2a that only strategic knowledge and not conceptual knowledge was a statistically significant predictor of the comprehensive diagnostic score. However, in bivariate correlation analyses, both types of knowledge displayed a medium correlation with the comprehensive diagnostic score and a medium correlation with each other. Thus, the non-significance of conceptual knowledge as a predictor might be due to its medium-level correlation with strategic knowledge (*r* = 0.55) and the shared variance of both variables. However, the amount of shared variance was acceptable, as highlighted by the reported variance inflation index.

Contrary to our expectations, there was mixed evidence for the relationship between professional knowledge and diagnostic accuracy. For one thing, there were significant bivariate correlations between conceptual and strategic knowledge and diagnostic accuracy (see Table 2). For another thing, both types of professional knowledge together did not predict the narrow diagnostic accuracy score in a regression and explained little variance (Model 4a). The non-significance of both knowledge types as predictors in the regression model could potentially be caused by their medium-level correlation. However, as previously mentioned, the shared variance between conceptual and strategic knowledge was acceptable. The small amount of explained variance discovered in the reported regressions for diagnostic accuracy can also be explained by looking at expertise development theory. Because the participants in our study were in their third to fifth year of medical school, it is reasonable to assume that they were still in or at the end of the initial stage of expertise development (Boshuizen & Schmidt, 1992; Evans & Patel, 1989). As the participants also had little experience in treating patients, it is likely that they possessed only a few illness scripts, and processes of knowledge integration and reorganization were not yet advanced. This lacking integration and reorganization of knowledge could have impeded participants’ application of their knowledge in diagnosing.

#### Are the diagnostic activities an embodiment of knowledge?

We also analyzed whether the diagnostic activities can be considered merely an embodiment of knowledge—or whether diagnostic activities can contribute to diagnostic success beyond prior knowledge. For the comprehensive diagnostic score and diagnostic accuracy score, hierarchical regressions demonstrated that the diagnostic activities added a significant amount of explained variance to that explained by participants’ professional knowledge ($\Delta$R^2^ = 0.21 resp. $\Delta$R^2^ = 0.20). This result provides preliminary evidence that diagnostic activities make a unique contribution to diagnostic success and are thus more than merely an embodiment of knowledge. There are two major possible mechanisms explaining this finding. First, the quality with which the diagnostic activities (i.e., hypothesis generation, evidence generation, and evidence evaluation in this study) were performed may have increased the medical students’ diagnostic success. Second, engagement in diagnostic activities with virtual patients may have helped the medical students access, activate or even generate relevant knowledge (i.e., learn) that they then implicitly applied in diagnosing.

### Limitations

One limitation is that clinical reasoning was explored in our history-taking study only with virtual patients. These virtual patients simulated history-taking through selecting brief video clips and tapped into diagnostic processes using instruments which led to a short pause in history-taking. These two characteristics may have evoked a process of reflectively diagnosing (Evans, 2008). If clinical reasoning is studied in history-taking contexts by using standardized patients, participants take part in conversations with actors. History-taking then happens in real time but diagnostic processes are subject to actors’ varying performance and biases from raters evaluating performance (Swanson & van der Vleuten, 2013). These points illustrate that virtual patients as well as standardized patients come with particular biases and advantages. We believe that the use of virtual patients as sole assessment method was justified but that our findings should be generalized to diagnosing real patients only with caution.

Other limitations have to do with the used instruments that captured the diagnostic process. The history-taking process modeled consisted of sequential steps, in which hypothesis generation and evidence generation were assessed before evidence evaluation was evaluated. Consequently, the success in later sequential steps of the diagnostic process depended to some extent on earlier diagnostic processes. Frameworks of clinical reasoning based on problem-solving theory (Elstein et al., 1978, 1990; Heitzmann et al., 2019) also assume that diagnostic processes influence each other. Nevertheless, it should be emphasized that real diagnostic situations rarely unfold sequentially and that clinicians may use different diagnostic processes at different points in time. Moreover, a newly-developed instrument was used to measure evidence evaluation. This instrument did only capture a part of the full process of evidence evaluation that takes place during history-taking and external validity evidence for this instrument was lacking. Not finding associations of evidence evaluation with diagnostic accuracy could be a result of the used instrument.

## Conclusions

We conducted a study assessing medical students’ clinical reasoning with virtual patients to examine to what extent knowledge and the diagnostic process, as operationalized by diagnostic activities, contribute to successful diagnosing. Our results provide support for clinical reasoning theories that conceptualize clinical reasoning as encompassing both process-related and knowledge-related aspects. Moreover, we found that the diagnostic activities learners engaged in made a unique contribution to diagnostic success, even when knowledge was considered. This result supports the view that the diagnostic process is—or can be—more than merely an embodiment of knowledge. There were two major possible mechanisms explaining this finding. First, the quality with which the diagnostic activities were performed may have increased the medical students’ diagnostic success. Second, engaging in diagnostic activities like generating hypotheses and evidence may have helped the medical students access, activate or generate relevant knowledge. Also, the reported findings suggest that diagnostic activities could potentially serve as a starting point for providing effective instructional support with cognitively-stimulating interventions.

## Author contributions

MCF contributed to the conceptualization and design of the study, gathered and analyzed the data, and created the first draft. NH contributed to the conceptualization and design of the study, advised on statistical analyses, and critically revised the article. VR contributed to the conceptualization and design of the study, advised on statistical analyses, and critically revised the article. MS conceptualized and designed the study, acquired funding, advised on statistical analyses, and critically revised the article. FF conceptualized and designed the study, acquired funding, advised on statistical analyses, and critically revised the article. MRF conceptualized and designed the study, acquired funding, advised on statistical analyses, and critically revised the article. All authors approved the final manuscript for publication and agreed to be accountable for all aspects of the work.

## Supplementary Information

Below is the link to the electronic supplementary material.

Supplementary file1 (DOCX 86 KB)
