# Supplementary material for: Diagnosing virtual patients: the interplay between knowledge and diagnostic activities
Source: Adv Health Sci Educ Theory Pract. 2023 Apr 13;28(4):1245–64. doi: 10.1007/s10459-023-10211-4 (PMC10099021; doi:10.1007/s10459-023-10211-4)
Supplement: Supplementary file 1 — Supplementary file1 (DOCX 86 KB) [file 10459_2023_10211_MOESM1_ESM.docx]

## Appendices

**Appendix S1. History-taking questions**

The history-taking menu has the following categories *main symptoms* (MS), *prior history* (PH), *allergies and medication* (AM), *social and family history* (SF), and *system review* (SR). The categories were adapted from Bornemann (2016).

*Table 1* *History-taking questions*

| **Category** | **Code** | **Example** |
| --- | --- | --- |
| Main symptoms | MS | Do you experience the complaints for the first time? |
| Prior history | PH | Do you know of any pre-existing conditions? |
| Allergies and medication | AM | Do you frequently have infections against which you take antibiotics? |
| Social and family history | SF | Have your parents or other relatives of your family passed away at a rather young age? |
| System review | SR | Has your weight changed within the last weeks? |

An overview of all 69 included questions is available in Fink et al. (2021).

Sources

Bornemann, B. (2016). *Dokumentationsbögen der Inneren Medizin und der Chirurgie für Anamnese und körperliche Untersuchung für die studentische Lehre in Deutschland*  (Diss., Institut für Didaktik und Ausbildungsforschung in der Medizin der Ludwig-Maximilians-Universität München). Retrieved from <https://edoc.ub.uni-muenchen.de/19166/>

Fink, M. C., Reitmeier, V., Stadler, M., Siebeck, M., Fischer, F., Fischer, M. R. (2021). Assessment of diagnostic competences with standardized patients versus virtual patients: Experimental study in the context of history taking. *Journal of Medical Internet Research*, *23*(3), e21196. https://doi.org/10.2196/21196

## Appendix S2. Principal component analysis for the comprehensive diagnostic score

| *Table 1 Component loadings* | | | | | | | | | | | | | | | |  |  |
| --- | --- | --- | --- | --- | --- | --- | --- | --- | --- | --- | --- | --- | --- | --- | --- | --- | --- |
|  | | | | | | **Component** | | | | | |  | | | |  |  |
|  | | | | | | **1** | | | | | | **Uniqueness** | | | |  |  |
| Diagnostic accuracy | | | | |  | 0.75 | | | | |  | 0.44 | | |  |  |  |
| Treatment selected | | | | |  | 0.78 | | | | |  | 0.39 | | |  |  |  |
| Expected findings in a physical examination | | | | |  | 0.74 | | | | |  | 0.45 | | |  |  |  |
| Diagnostic measures taken for medical clarification | | | | |  | 0.85 | | | | |  | 0.28 | | |  |  |  |
| 'varimax' rotation was used | | | | | | | | | | | | | | | |  |  |
|  | | | | | | | | | | | | | | | |  |  |
| *Table 2 Component statistics summary* | | | | | | | | | | | | | | | | | |
|  |  | | |  | | |  | | |  | | |  |  | | |  |
| **Component** | | **SS Loadings** | | | | | | **% of Variance** | | | | | | **Cumulative %** | | | |
| 1 |  | | 2.44 | | | |  | | 61.09 | | | |  | 61.09 | | |  |

| *Table 3 Initial Eigenvalues* | | | | | | | |
| --- | --- | --- | --- | --- | --- | --- | --- |
|  |  |  |  |  |  |  |  |
| **Component** | | **Eigenvalue** | | **% of Variance** | | **Cumulative %** | |
| 1 |  | 2.44 |  | 61.09 |  | 61.09 |  |
| 2 |  | 0.69 |  | 17.14 |  | 78.22 |  |
| 3 |  | 0.53 |  | 13.13 |  | 91.36 |  |
| 4 |  | 0.35 |  | 8.64 |  | 100.00 |  |
|  | | | | | | | |

 
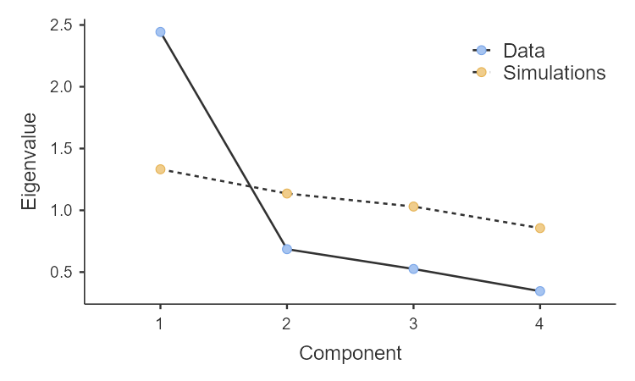

Scree Plot

## Appendix S3. Diagnoses of the virtual patients

*Table 1* *Diagnoses of the virtual patients*

|  |  |  | **Case number in the study** | **Case number in the paper** | **Diagnosis** | **Patient characteristics** | **Patient name** |
| --- | --- | --- | --- | --- | --- | --- | --- |
|  |  |  | 1 | 1 | Hypertrophic cardiomyopathy | 25 years, male | Mr. Albrecht |
|  |  |  | 2 | 2 | Pneumonia | 55 years, female | Ms. Klein |
|  |  |  | 3 | _ | Pulmonary embolism in case of prostate cancer | 70 years, male | Mr. Wagner |
|  |  |  | 4 | 3 | Pulmonary embolism with coagulation disorder | 35 years, female | Ms. Aimüller |
|  |  |  | 5 | _ | Heart insufficiency with thoracic aortic aneurysm | 65 years, female | Ms. Bircher |
|  |  |  | 6 | 4 | Panic attack | 45 years, male | Mr. Lehner |

We discovered floor effects on the diagnostic success measures diagnostic accuracy, and treatment selected (see Table 2). Therefore, these cases were excluded from our study.

*Table 2 Descriptive statistics of the diagnostic success measures of the excluded cases*

|  | **Mr. Wagner** | **Ms. Bircher** |
| --- | --- | --- |
| Diagnostic accuracy | 0.04 (0.14) | 0.08 (0.23) |
| Treatment selected | 0.01 (0.10) | 0.09 (0.28) |
| Expected findings in a physical examination | 0.38 (0.16) | 0.42 (0.20) |
| Diagnostic measures taken for medical clarification | 0.12 (0.11) | 0.43 (0.19) |

## Appendix S4. Control analysis for data collection

Table 1 Descriptives for professional knowledge, diagnostic activities, and diagnostic success measures across both data collection groups and results of independent samples t-tests

| **Variable** | **Web-based data collection *M* (*SD*)** | **Lab-based data collection *M* (*SD*)** | ***t*** | ***df*** | ***p*** |
| --- | --- | --- | --- | --- | --- |
| Conceptual knowledge | 0.50 (0.13) | 0.62 (0.13) | -4.36 | 104 | < .001 |
| Strategic knowledge | 0.47 (0.14) | 0.58 (0.13) | -3.85 | 104 | < .001 |
| Hypothesis generation | 0.36 (0.19) | 0.34 (0.18) | 0.59 | 104 | .554 |
| Evidence generation | 0.41 (0.12) | 0.51 (0.11) | -3.53 | 104 | < .001 |
| Evidence evaluation | 0.48 (0.11) | 0.50 (0.12) | -0.89 | 104 | .374 |
| Diagnostic accuracy | 0.39 (0.21) | 0.54 (0.23) | -3.11 | 104 | .002 |
| Comprehensive diagnostic score | -0.14 (0.75) | 0.34 (0.73) | -2.99 | 104 | .004 |

Comprehensive diagnostic score was a normalized z-score ranging from -3 to +3. Scale range for all other variables: 0-1. Note that the scores for hypothesis generation, evidence generation, evidence evaluation, diagnostic accuracy, and comprehensive diagnostic score were aggregated over multiple virtual patients.

## Appendix S5. Regression analyses, including data collection as a factor

*Table 1 Regression analyses for the comprehensive diagnostic score as outcome, including
the data collection method as a categorical variable*

| **Predictor** | | **b** | **ß** | **p** |
| --- | --- | --- | --- | --- |
| Intercept | -3.10 | |  | < .001 |
| Data collection | 0.96 | | 0.22 | .303 |
| Conceptual knowledge | 1.01 | | 0.18 | .111 |
| Strategic knowledge | 0.77 | | 0.14 | .240 |
| Hypothesis generation | 1.21 | | 0.29 | .002 |
| Evidence evaluation | 1.88 | | 0.28 | .006 |
| Evidence generation | 1.79 | | 0.30 | .006 |
| Conceptual knowledge ✻ Data collection | -0.04 | | -0.01 | .978 |
| Strategic knowledge ✻ Data collection | -0.02 | | -0.00 | .986 |
| Hypothesis generation ✻ Data collection | -0.07 | | -0.02 | .934 |
| Evidence generation ✻ Data collection | -0.95 | | -0.16 | .460 |
| Evidence evaluation ✻ Data collection | -0.64 | | -0.09 | .629 |

F(11, 94) = 6.30, p < .001, *R*² = .42

*Table 2 Regression analyses for diagnostic accuracy as outcome, including
the data collection method as a categorical variable*

| **Predictor** | | **b** | | **ß** | **p** |
| --- | --- | --- | --- | --- | --- |
| Intercept | -0.03 | |  | | .832 |
| Data collection | 0.23 | | 0.62 | | .433 |
| Conceptual knowledge | 0.17 | | 0.11 | | .387 |
| Strategic knowledge | 0.05 | | 0.03 | | .803 |
| Hypothesis generation | 0.41 | | 0.34 | | .001 |
| Evidence evaluation | 0.12 | | 0.06 | | .566 |
| Evidence generation | 0.25 | | 0.14 | | .213 |
| Conceptual knowledge ✻ Data collection | -0.25 | | -0.15 | | .564 |
| Strategic knowledge ✻ Data collection | 0.14 | | 0.09 | | .745 |
| Hypothesis generation ✻ Data collection | 0.43 | | 0.35 | | .116 |
| Evidence generation ✻ Data collection | -0.27 | | -0.15 | | .508 |
| Evidence evaluation ✻ Data collection | -0.13 | | -0.06 | | .761 |

F(11, 94) = 4.09, p < .001, *R*² = .32

## Appendix S6. Regression analyses based on participation in the module respiratory diseases

**The contribution of diagnostic activities and professional knowledge to the comprehensive diagnostic score**

*Table 1 Regression analyses for comprehensive diagnostic score as outcome for the participants who did not take part in the module respiratory diseases*

| **Predictor** | ***b*** | | ***ß*** | ***ß* 95% CI** | ***p*** | ***Model test and fit*** | |
| --- | --- | --- | --- | --- | --- | --- | --- |
| Model 1 |  | |  |  |  | | *F*(3, 30) = 3.61, *p* = .024 |
| Intercept | -2.16 | |  |  | .003 | | *R^2^* = .27 |
| Hypothesis generation | 1.43 | | 0.39 | [0.05, 0.72] | .024 | | Adj. *R*^2^ = .19 |
| Evidence generation | 1.20 | | 0.23 | [-0.10, 0.56] | .169 | |  |
| Evidence evaluation | 1.89 | | 0.28 | [-0.04, 0.61] | .081 | |  |
| Model 2a |  | |  |  |  | | *F*(2, 31) = 2.98, *p* = .065 |
| Intercept | -1.28 | |  |  | .037 | | *R^2^* = .16 |
| Conceptual knowledge | | 0.62 | 0.09 | [-0.27, 0.45] | .608 | | Adj. *R*^2^ = .11 |
| Strategic knowledge | | 1.67 | 0.36 | [0.00, 0.72] | .047 | |  |
| Model 2b | |  |  |  |  | | *F*(5, 28) = 3.16, *p* = . 022 |
| Intercept | | -2.87 |  |  | .002 | | *R^2^* =.36 |
| Conceptual knowledge | | 1.16 | 0.17 | [-0.17, 0.51] | .313 | | Adj. *R*^2^ = .25 |
| Strategic knowledge | | 1.20 | 0.26 | [-0.13, 0.65] | .179 | |  |
| Hypothesis generation | | 1.23 | 0.33 | [0.00, 0.66] | .048 | |  |
| Evidence generation | | 0.24 | 0.05 | [-0.33, 0.43] | .804 | |  |
| Evidence evaluation | | 1.98 | 0.30 | [-0.02, 0.62] | .067 | |  |

Model 1 is a multiple regression containing diagnostic activities variables. Model 2 is a hierarchical regression consisting of knowledge variables in Model 2a and knowledge and diagnostic activities in Model 2b. *b* represents unstandardized regression weights. *ß* represents standardized regression weights. CI = confidence interval.

*Table 2 Regression analyses for comprehensive diagnostic score as outcome for the participants who took part in the module respiratory diseases*

| **Predictor** | ***b*** | | ***ß*** | ***ß* 95% CI** | ***p*** | ***Model test and fit*** | |
| --- | --- | --- | --- | --- | --- | --- | --- |
| Model 1 |  | |  |  |  | | *F*(3, 59) = 9.71, *p* < .001 |
| Intercept | -2.15 | |  |  | <.001 | | *R^2^* = .33 |
| Hypothesis generation | 1.04 | | 0.26 | [0.04, 0.47] | .023 | | Adj. *R*^2^ = .30 |
| Evidence generation | 2.30 | | 0.35 | [0.14, 0.57] | .002 | |  |
| Evidence evaluation | 1.78 | | 0.27 | [0.06, 0.49] | .014 | |  |
| Model 2a | |  |  |  |  | | *F*(2, 60) = 5.75, *p* = .005 |
| Intercept | | -1.07 |  |  | .007 | | *R^2^* = .16 |
| Conceptual knowledge | | 1.05 | 0.21 | [-0.09, 0.51] | .164 | | Adj. *R*^2^ = .13 |
| Strategic knowledge | | 1.24 | 0.23 | [-0.07, 0.53] | .125 | |  |
| Model 2b | |  |  |  |  | | *F*(5, 57) = 7.45, *p* < .001 |
| Intercept | | -2.60 |  |  | <.001 | | *R^2^* =.40 |
| Conceptual knowledge | | 0.77 | 0.15 | [-0.11, 0.42] | .247 | | Adj. *R*^2^ = .34 |
| Strategic knowledge | | 0.83 | 0.16 | [-0.13, 0.44] | .279 | |  |
| Hypothesis generation | | 1.17 | 0.29 | [0.07, 0.51] | .010 | |  |
| Evidence generation | | 1.65 | 0.25 | [0.03, 0.48] | .029 | |  |
| Evidence evaluation | | 1.46 | 0.23 | [0.01, 0.44] | .042 | |  |

Model 1 is a multiple regression containing diagnostic activities variables. Model 2 is a hierarchical regression consisting of knowledge variables in Model 2a and knowledge and diagnostic activities in Model 2b. *b* represents unstandardized regression weights. *ß* represents standardized regression weights. CI = confidence interval.

**The contribution of diagnostic activities and professional knowledge to diagnostic accuracy**

*Table 3 Regression analyses for diagnostic accuracy as outcome for the participants who did not take part in the module respiratory diseases*

| **Predictor** | ***b*** | ***ß*** | ***ß* 95% CI** | ***p*** | ***Model test and fit*** |
| --- | --- | --- | --- | --- | --- |
| Model 3 |  |  |  |  | *F*(3, 30) = 1.69, *p* = .191 |
| Intercept | 0.26 |  |  | .222 | *R^2^* = .14 |
| Hypothesis generation | 0.42 | 0.39 | [0.03, 0.75] | .034 | Adj. *R*^2^ = .06 |
| Evidence generation | 0.10 | 0.06 | [-0.29, 0.42] | .717 |  |
| Evidence evaluation | -0.15 | -0.08 | [-0.43, 0.27] | .647 |  |
| Model 4a |  |  |  |  | *F*(2, 31) = 0.34, *p* = .716 |
| Intercept | 0.27 |  |  | .153 | *R^2^* = .02 |
| Conceptual knowledge | 0.05 | 0.03 | [-0.36, 0.41] | .889 | Adj. *R*^2^ = -.04 |
| Strategic knowledge | 0.18 | 0.13 | [-0.25, 0.52] | .482 |  |
| Model 4b |  |  |  |  | *F*(5, 28) = 1.07, *p* = .400 |
| Intercept | 0.21 |  |  | .452 | *R^2^* = .16 |
| Conceptual knowledge | 0.05 | 0.03 | [-0.36, 0.41] | .892 | Adj. *R*^2^ = .01 |
| Strategic knowledge | 0.18 | 0.14 | [-0.31, 0.58] | .533 |  |
| Hypothesis generation | 0.40 | 0.36 | [-0.01, 0.74] | .057 |  |
| Evidence generation | -0.03 | -0.02 | [-0.45, 0.42] | .937 |  |
| Evidence evaluation | -0.16 | -0.08 | [-0.45, 0.28] | .646 |  |

Model 3 is a multiple regression containing diagnostic activities variables. Model 4 is a hierarchical regression, consisting of knowledge variables in Model 4a and knowledge and diagnostic activities in Model 4b. *b* represents unstandardized regression weights. *ß* represents standardized regression weights. CI = confidence interval. * *p* < .05, ** *p* < .01, *** p < .001

*Table 4 Regression analyses for diagnostic accuracy as outcome for the participants who took part in the module respiratory diseases*

| **Predictor** | ***b*** | ***ß*** | ***ß* 95% CI** | ***p*** | ***Model test and fit*** |
| --- | --- | --- | --- | --- | --- |
| Model 3 |  |  |  |  | *F*(3, 59) = 7.36, *p* < .001 |
| Intercept | 0.01 |  |  | .940 | *R^2^* = .27 |
| Hypothesis generation | 0.54 | 0.43 | [0.21, 0.66] | <.001 | Adj. *R*^2^ = .24 |
| Evidence generation | 0.34 | 0.17 | [-0.05, 0.39] | .135 |  |
| Evidence evaluation | 0.24 | 0.12 | [-0.11, 0.35] | .299 |  |
| Model 4a |  |  |  |  | *F*(2, 60) = 2.07, *p* = .135 |
| Intercept | 0.25 |  |  | .048 | *R^2^* = .06 |
| Conceptual knowledge | 0.35 | 0.23 | [-0.09, 0.54] | .159 | Adj. *R*^2^ = .03 |
| Strategic knowledge | 0.07 | 0.04 | [-0.28, 0.36] | .796 |  |
| Model 4b |  |  |  |  | *F*(5, 57) = 5.12 *p* < .001 |
| Intercept | -0.10 |  |  | .546 | *R^2^* = .31 |
| Conceptual knowledge | 0.23 | 0.15 | [-0.13, 0.43] | .290 | Adj. *R*^2^ = .25 |
| Strategic knowledge | 0.13 | 0.08 | [-0.23, 0.38] | .606 |  |
| Hypothesis generation | 0.57 | 0.45 | [0.22, 0.68] | < .001 |  |
| Evidence generation | 0.20 | 0.10 | [-0.14, 0.34] | .410 |  |
| Evidence evaluation | 0.17 | 0.09 | [-0.15, 0.32] | .457 |  |

Model 3 is a multiple regression containing diagnostic activities variables. Model 4 is a hierarchical regression, consisting of knowledge variables in Model 4a and knowledge and diagnostic activities in Model 4b. *b* represents unstandardized regression weights. *ß* represents standardized regression weights. CI = confidence interval. * *p* < .05, ** *p* < .01, *** p < .001
